# Supplementary material for: Association of perioperative serum carcinoembryonic antigen level and recurrence in low-risk stage IIA colon cancer
Source: PLoS One. 2021 Jun 9;16(6):e0252566. doi: 10.1371/journal.pone.0252566 (PMC8189445; doi:10.1371/journal.pone.0252566)
Supplement: Attachment — Submitted filename: Response to Reviewers.docx [file pone.0252566.s002.docx]

Reviewer #1

The reference has been modified to put it at the end of the sentence but before the'period'. Attached to the discussion are discussions about the role of adjuvant therapy with or without surgery at different stages of the CRC.

Reviewer #2

Additionally, Tables 7 and 8 were created according to the presence or absence of diabetes. When only patients without diabetes were studied, statistically significant results were obtained in group L vs H, and when only patients with diabetes were studied, there were no statistically significant factors. Therefore, for low-risk stage IIA colon cancer patients, when the perioperative serum CEA level is higher than a certain level, it can be confirmed that the recurrence and mortality rate are higher than those without.

Table 7. Uni- and multivariate analysis of risk factors associated with recurrence using Cox regression model (DM-)

| Factors | Univariate | Multivariate |  |  |
| --- | --- | --- | --- | --- |
|  | *P* | HR | 95% CI | *P* |
| Age (<70 vs. ≥70 years) | 0.121 |  |  |  |
| Gender (female vs. Male) | 0.709 |  |  |  |
| BMI (<25 vs. ≥25 kg/m2) | 0.643 |  |  |  |
| preCEA (<5 vs. ≥5 ng/mL) | 0.152 |  |  |  |
| postCEA (<5 vs. ≥5 ng/mL) | 0.596 |  |  |  |
| Group (L vs.H) | 0.021 | 2.25 | 1.13-4.51 | 0.021 |
| Tumor site (Right vs. Left) | 0.118 |  |  |  |
| Underlying disease | 0.193 |  |  |  |
| Smoking | 0.928 |  |  |  |
| Alcohol | 0.996 |  |  |  |
| Complication | 0.138 |  |  |  |
| Histology (WD vs. MD) | 0.629 |  |  |  |

preCEA: preoperative carcinoembryonic antigen, WD: well differentiated, MD: moderately differentiated, HR: hazard ratio, 95% CI: 95% confidence interval

Table 8. Uni- and multivariate analysis of risk factors associated with recurrence using Cox regression model (DM+)

| Factors | Univariate | Multivariate |  |  |
| --- | --- | --- | --- | --- |
|  | *P* | HR | 95% CI | *P* |
| Age (<70 vs. ≥70 years) | 0.084 | 0.39 | 0.13-1.12 | 0.084 |
| Gender (female vs. Male) | 0.493 |  |  |  |
| BMI (<25 vs. ≥25 kg/m2) | 0.169 |  |  |  |
| preCEA (<5 vs. ≥5 ng/mL) | 0.867 |  |  |  |
| postCEA (<5 vs. ≥5 ng/mL) | 0.778 |  |  |  |
| Group (L vs.H) | 0.417 |  |  |  |
| Tumor site (Right vs. Left) | 0.533 |  |  |  |
| Underlying disease | - |  |  |  |
| Smoking | 0.117 |  |  |  |
| Alcohol | 0.912 |  |  |  |
| Complication | 0.478 |  |  |  |
| Histology (WD vs. MD) | 0.515 |  |  |  |

preCEA: preoperative carcinoembryonic antigen, WD: well differentiated, MD: moderately differentiated, HR: hazard ratio, 95% CI: 95% confidence interval
